# Exploring the Stress Impact in the Paternal Germ Cells Epigenome: Can Catecholamines Induce Epigenetic Reprogramming?

**DOI:** 10.3389/fendo.2020.630948

**Published:** 2021-02-19

**Authors:** Candela R. González, Betina González

**Affiliations:** ^1^ Centro de Estudios Biomédicos Básicos, Aplicados y Desarrollo (CEBBAD), Universidad Maimónides, Buenos Aires, Argentina; ^2^ Instituto de Investigaciones Farmacológicas (Universidad de Buenos Aires–Consejo Nacional de Investigaciones Científicas y Técnicas), Ciudad Autónoma de Buenos Aires, Buenos Aires, Argentina

**Keywords:** male germ cells, epigenetics, catecholamines, dopamine receptor, adrenergic receptor

## Abstract

Spermatogenesis is characterized by unique epigenetic programs that enable chromatin remodeling and transcriptional regulation for proper meiotic divisions and germ cells maturation. Paternal lifestyle stressors such as diet, drug abuse, or psychological trauma can directly impact the germ cell epigenome and transmit phenotypes to the next generation, pointing to the importance of epigenetic regulation during spermatogenesis. It is established that environmental perturbations can affect the development and behavior of the offspring through epigenetic inheritance, including changes in small non-coding RNAs, DNA methylation, and histones post-translational modifications. But how male germ cells react to lifestyle stressors and encode them in the paternal epigenome is still a research gap. Most lifestyle stressors activate catecholamine circuits leading to both acute and long-term changes in neural functions, and epigenetic mechanisms show strong links to both long-term and rapid, dynamic gene expression regulation during stress. Importantly, the testis shares a molecular and transcriptional signature with the brain tissue, including a rich expression of catecholaminergic elements in germ cells that seem to respond to stressors with similar epigenetic and transcriptional profiles. In this minireview, we put on stage the action of catecholamines as possible mediators between paternal stress responses and epigenetic marks alterations during spermatogenesis. Understanding the epigenetic regulation in spermatogenesis will contribute to unravel the coding mechanisms in the transmission of the biological impacts of stress between generations.

## Introduction

Stress is a phenomenon fundamental to survival, in which complex and timely physiological and behavioral responses, allow the individual to adapt to the dynamic challenges of the environment and restore body homeostasis (1). The stress response is activated by the sympathoneural and sympathoadrenomedullary systems that secrete catecholamines, which in turn activates the hypothalamus-pituitary-adrenal (HPA) axis to secrete glucocorticoids. The interplay between these circuits systemically promotes metabolic and behavioral changes that are transient and adaptive; however, the prolonged sympathetic stimulation and increased glucocorticoid levels during chronic stress has been associated with long-lasting maladaptive responses (2). In the last years, the discovery of epigenetic mechanisms that could alter the sperm information and transmit stress-related phenotypes to the offspring was a huge breakthrough for the male reproduction field (3). It is now clear that pathophysiological effects of stress are not confined to the individual, but stress can affect the first generation offspring and even extend across multiple generations through the epigenetic reprogramming of male germ cells (4–6).

**Figure 1:**
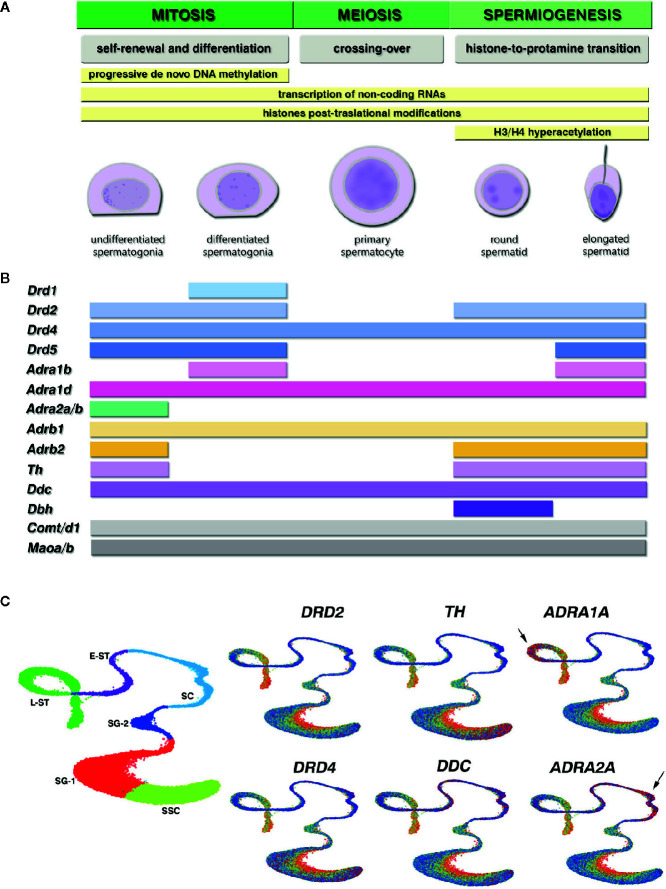
Epigenetic programming during spermatogenesis and expression of the catecholaminergic system components in different germ cell stages of the mouse and human spermatogenesis. **(A)** Curated stages of spermatogenesis available at the Gene Expression Omnibus (GEO) database. Yellow bars show the epigenetic mechanisms that contribute to key functional events (gray) characteristic of each cell stage during mitosis, meiosis and spermiogenesis, where environmental stressors are most able to reprogram epigenetic marks. **(B)** Gene expression profile in mouse spermatogenic cells, obtained from cell-stage specific RNA-seq and single-cell RNA-seq datasets GEO database. The colored bars depict at which cell stage all datasets reported positive transcript detection for the catecholamine receptors and enzymes genes [mean RNA-seq RPKM/FPKM > 0.05 and single-cell RNA-seq log(sum normalized expression in the cluster + 1) > 1.2]. The picture shows: dopamine receptors from the D1 family *Drd1*, *Drd5*, and D2 family *Drd2*, *Drd4*; adrenergic receptors from the α1-subtype *Adra1b*, *Adra1d*, α2-subtype *Adra2a*, *Adra2b*, and β-subtype *Adrb1*, *Adrb2*; dopamine synthesizing enzymes tyrosine hydroxylase (*Th*) and aromatic-L-aminoacid-decarboxylase (*Ddc*); nor-epinephrine converting enzyme dopamine-beta-hydroxylase (*Dbh*); catecholamine eliminating enzymes catechol-O-methyl-transferase *Comt, Comtd1*, and monoamine oxydase *Maoa*, *Maob*. Genes within the same family that showed coincident expression pattern are depicted together. We found no consistent expression of *Drd3*, *Adra1a*, *Adra2c*, and *Adrb3* between datasets for any cell stage. **(C)** Gene expression visualization for selected genes in the Human Testis integrated dataset, available at the UCSC cell browser (https://testis.cells.ucsc.edu/), that combines the currently available human testis single-cell RNA-seq datasets (8). We selected Uniform Manifold Approximation and Projection (UMAP) for dimension reduction to plot the quantitative gene expression pattern in increasing blue-to-red gradient color code. Left plot shows the cells clusters distribution: SSC, spermatogonial stem cells; SG-1, spermatogonia cluster 1, SG-2, spermatogonia cluster 2 committed to meiosis; SC, spermatocytes; E-ST, early spermatids; L-ST, late spermatids. Arrows indicate specific high red detection for *ADRA1A* in late spermatids, and *ADRA2A* in spermatocytes, in the Human Testis integrated dataset that was not detected in the mouse datasets.

Epigenetic information in male germ cells involves changes in small non-coding RNAs, paternal DNA methylation and histones post-translational modifications (PTMs) patterns that modulate gene expression in response to basal transcriptional programs and environmental stressors (7). Spermatogenesis is characterized by a unique epigenetic program that enables chromatin remodeling to protect paternal DNA, and a fine transcriptional regulation required for proper meiotic divisions and sperm maturation (**Figure 1A**). Within male germ cells, changes in epigenetic states are critical for the silencing of transposable elements, paternal genes imprinting, several aspects of meiosis, post-meiotic gene silencing and DNA compaction (9). Once meiosis is completed, the hyperacetylation of histones H3/H4 initiates the essential histone-to-protamine replacement that enables chromatin remodeling and compaction during spermiogenesis (10–13). It was recently found that protamines also carry several PTMs (14), suggesting the existence of a “protamine code” that could be involved in the epigenetic mechanisms that control the incorporation of maternal histones to paternal DNA after fertilization (15). Interestingly, a small percentage of histones, 10–15% in humans and 1–8% in mice, are retained in sperm chromatin (16–18) being their location associated with promoters of functional genes during spermatogenesis and at early embryonic development (7, 10, 11, 19). Active transcription in male germ cells takes place in the stages of spermatogonia, spermatocytes, and round spermatids, where epigenetic patterns are established (3, 7). Therefore, these spermatogenic stages are considered *windows of vulnerability* where the paternal epigenome could be reprogrammed by environmental stressors (20).

The catecholamine and glucocorticoid systems elicit dynamic molecular adaptations of the central nervous system (CNS) during stress, contributing to long-term consequences on physiological and behavioral traits that can be transmitted to the offspring (21–23). So far, a clear link has been demonstrated between glucocorticoid receptor (GR) activation by corticosterone administration and increased levels of microRNAs and DNA methylation in sperm (24, 25). However, the catecholamines specific impact on the paternal epigenome is still a research gap. Here, the question arises: can germ cells react to catecholamines signaling and encode it in different epigenetic marks in the paternal genome? To tackle this question, we discuss how the testis shares a molecular and transcriptional signature with the brain tissue, including a rich expression of catecholaminergic elements in germ cells that seem to respond to stressors with similar epigenetic and transcriptional profiles. In this minireview, we put on stage the action of catecholamines as potential regulators of the *stress epigenetic memory encoding* in germ cells.

### Epigenetic Mechanisms Related to Catecholamines Signaling

Most lifestyle stressors activate catecholamine circuits leading to both acute and long-term changes in neural functions, and epigenetic mechanisms are strongly suspected in both long-term and rapid, dynamic gene expression regulation during stress (26). Environmental stressors rapidly activate the sympathetic system and induce changes in the production and secretion of neurotransmitters and stress hormones including the catecholamines dopamine (DA) and nor-epinephrine (NE)/epinephrine (E), which orchestrate central and peripheral downstream effects that enable the behavioral and systemic response to stress. Dopaminergic tissues express the rate-limiting tyrosine hydroxylase (TH) and the aromatic-L-aminoacid-decarboxylase (AADC or DDC), that convert L-tyrosine to DA. Adrenergic tissues also express dopamine-beta-hydroxylase (DBH) that further converts DA to NE, and the phenylethanolamine-N-methyltransferase (PNMT) converts NE to E in the adrenal medulla (27, 28). The stress response rapidly release catecholamines that activate the HPA axis, which in turn induces both neuronal and non-neuronal expression of TH, DDC and DBH, increasing central and peripheral catecholamine production (27, 28). Catecholamines bind to G protein-coupled receptors (GPCRs) that signal though cAMP and Ca^2+^ second messengers. DA effects are mediated by two types of DA receptors (DRDs): D1-like (D1, D5), that are coupled to Gα_s_/Gα_olf_ and rise cAMP levels, and D2-like (D2, D3, D4) DRDs, that are coupled to Gα_i_ and blunt cAMP levels (29). NE/E effects are mediated by activation of three types and nine subtypes of adrenergic receptors (ADRs): alpha1- (α1a/c, α1b, α1d), coupled to Gα_q_ to increase cytoplasmic Ca^2+^ levels, alpha2- (α2a, α2b, α2c), coupled to Gα_i_, and beta- (β1, β2, β3) ADRs, coupled to Gα_s_ (30). Upon production and release, catecholamines are rapidly deactivated by the catabolic catechol-O-methyltransferase (COMT) and monoamine oxidase (MAO) enzymes (27, 28).

The regulation of DA and NE synthesis, release and signaling is extremely sensitive to environmental perturbations, affecting gene expression through epigenetic mechanisms in the brain (31–33). Catecholamine receptors downstream effects are related to the modulation of cAMP and Ca^2+^ levels, that control signaling effectors like protein kinases PKA and PKC, and calmodulin kinases such as CaMKII (29, 34, 35). Stress-induced overstimulation of Gα_s_-coupled D1-DRDs and β-ADRs was linked to increased oxidative stress and PKA recruitment of mitogen-activated protein kinases (MAPKs) (36–38), which translocate into the nucleus to phosphorylate and activate several transcription factors including the cAMP response element-binding protein (CREB), activator protein 1 (AP-1), transcription activator Elk-1, nuclear factor κB (NFκB) and H3 histones, to control target gene expression (34, 36, 37). So far, the best characterized stress-induced epigenetic effects involve increased H3 phosphoacetylation (26, 33) and changes in DNA and H3K9/K27 methylation patterns in target genes, affecting components of the stress system like corticotropin releasing hormone (CRH), GR and MAO-A, trophic factors like brain-derived neurotrophic factors (BDNF), as well as inflammation-, oxidative-, mitochondrial-, and endoplasmic reticulum stress-related genes (26, 36, 37, 39). Importantly, many stress-induced epigenetic effects depend on GR activation, a nuclear receptor that elicits both genomic and non-genomic changes in gene expression by direct binding to DNA, and by forming complexes with MAPKs (40, 41), and epigenetic effectors like chromatin modifying enzymes and microRNAs (42). Moreover, GR binds to TH, DBH, and PNMT promoters to increase gene expression and catecholamine production (27), and it was also show to control catecholamines receptors expression such as α1- and β-ADRs (43, 44). But if these stress mechanisms can also impact the testis and establish an epigenetic memory in germ cells has not been fully elucidated.

### Daddy Has Two Brains

The testis shares a close transcriptional and proteomic signature with the brain, as it was shown in tissue microarray and proteomic studies in human and mice (45–47). It is clear that the interstitial compartment behaves as a diffuse neuroendocrine (paraneuron) system, where most cell types share properties with neurons, including production of amine and peptide hormones/transmitters, and specific markers of neural determination (48, 49). Pioneer studies in the human and primate testis showed that the interstitial compartment, together with an extrinsic sympathetic innervation provided by the spermatic nerves (50, 51), has an intrinsic catecholaminergic input provided by neuron-like APUD (amine precursor uptake and decarboxylation) cells, that cooperate with the autonomous brain system to regulate tissue homeostasis (52–54).

The close relationship between brain and reproductive tissues can be traced even to the mature sperm. Recent work by Ramírez-Reveco et al. (55), postulated the original and controversial idea that sperm acrosome reaction includes several steps that recall the process of presynaptic secretion. The authors also proposed that the common embryonic origin between the testis and nervous system could explain the presence of “neural elements” in the sperm, including DA and NE transporters and receptors (56–58). Interestingly, recent evidences showed that the sex determining region on the Y chromosome (SRY) transcription factor, responsible for the differentiation of the bipotent gonadal ridge into the testis, behaves as a catecholaminergic program inductor that controls voluntary movement in adult brain dopaminergic areas (59, 60). SRY was found to potentiate DA synthesis and metabolism by binding to TH enhancer, and to increase DDC, DRD2, and MAO-A levels in dopaminergic neurons (59). Moreover, testosterone through androgen receptor activation was found to participate in the regulation of cortical DA neurotransmission, modulating TH levels, DA metabolism, and cognition in male rodents (61).

So far, the best characterized actions of catecholamines in the testis are related to their targets in the interstitial compartment and the control of steroidogenesis (52, 53, 62), whereas their possible direct effects in the seminiferous tubule have been sidelined. Despite research on specific catecholamine actions during spermatogenesis was neglected, there are well-documented examples of sympathomimetic drugs, e.g., cocaine, that affect the male germ cell epigenome (63–66). In the last years, evidence has emerged pointing to modifications in sperm DNA methylome in paternal stress models involving cocaine intake (64, 65). We and others have reported the effects of cocaine on specific H3/H4 PTMs related to altered epigenetic marking of BDNF in sperm (63), and to the silencing of gene transcription and the histone-to-protamine replacement through a DRD1-dependent mechanism in maturing sperm cells (66). Moreover, we showed for the first time that cocaine increases the testicular expression of TH (67) and induces a down-regulation of DRDs in mouse germ cells (66), similarly to the cocaine mechanism described in the brain. Interestingly, we found similar changes on germ cells H3/H4 PTMs after either DRD1-inhibitor or cocaine treatment, an effect known in the brain as the “inverted U-shaped DA response,” where stimulation above and below the optimal level will equally decrease function and have similar detrimental effects (68). To our knowledge, this was the first report that proposed a catecholaminergic pathway as a mechanism of epigenetic marks encoding in male germ cells. Despite all these data, it has not been investigated the cellular stage at which male germ cells may acquire catecholaminergic components and their potential role in paternal epigenome reprogramming.

### An Open Window for Catecholamine-Induced Epigenetic Reprogramming

It is actually established that environmental stressors may influence the male germline differently depending on timing (20). Each spermatogenic stage has a characteristic epigenetic landscape that enables the complex transcriptional programs that drive mitosis, meiosis and spermiogenesis, being susceptible to environmental interactions with their active epigenetic machinery (3) (**Figure 1A**). This provides evidence of the broad timing of plasticity during spermatogenesis. However, there are still research gaps concerning by which mechanisms and when in the germ cell maturation stage epigenetic marks can be programmed.

Research on catecholaminergic components expression during specific stages of spermatogenesis is scarce and sometimes inconclusive. To overcome the gaps in the literature and bring a complete scenario, we conducted a query at the Gene Expression Omnibus Database (GEO) for publicly available high-throughput RNA sequencing (bulk and single-cell RNA-seq) datasets in purified male germ cells populations. **Table 1** shows the details of the curated datasets. We analyzed the normalized expression reported for gene transcripts of interest in each study, and selected those genes that showed positive expression for each specific cell stage in all datasets (**Figure 1B**). We also analyzed the human cell-specific single-cell RNA-seq data in the Human Testis integrated dataset (8), available at UCSC cell browser portal (https://testis.cells.ucsc.edu/), that combines the currently available single-cell RNA-seq human testis datasets (**Figure 1C**). Overall, we detected expression for catecholaminergic receptors and metabolic enzymes throughout the mouse and human spermatogenesis, which suggest that male germ cells are both a source and a target of catecholamines.

**Table 1:** Datasets selected from the Gene Expression Omnibus Database (GEO).

Species	Method	Cell type	Dataset	Reference
Mouse	RNA-seq	Undifferentiated spermatogonia (Thy1+)	GSE49622	Hammoud et al. (69)
		Differentiated spermatogonia (Kit+)		
		Primary spermatocyte		
		Round spermatid		
Mouse	RNA-seq	Primary spermatocyte	GSE43717	Soumillon et al. (70)
		Round spermatid		
Mouse	RNA-seq	Differentiated spermatogonia (cKit+)	GSE89502	Maezawa et al. (71)
Mouse	RNA-seq	Undifferentiated spermatogonia (PLZF+ cKIT−)	GSE107124	La et al. (72)
Mouse	scRNA-seq	Undifferentiated spermatogonia (Gfra1+)	GSE112393	Green et al. (73)
		Primary spermatocyte		
		Round spermatid		
		Elongated spermatid		
Mouse	scRNA-seq	Undifferentiated spermatogonia (ID4-EGFP+)	GSE108974	Hermann et al. (74)

In the adult testis, undifferentiated spermatogonia stem cells (SSCs) must achieve a stable balance between self-renewal and differentiated spermatogonia, which commit to meiosis and give rise to primary spermatocytes. As it is shown in **Figure 1B**, differentiated spermatogonia are the spermatogenic stages that show most expression of cathecolaminergic receptors mRNAs, including DRDs *Drd1/5*, *Drd2/4*, α1-ADRs *Adra1b/d*, α2-ADRs *Adra2a/b*, and β−ADRs *Adrb1/2.* Catecholaminergic receptors mRNAs expression declines at meiotic stages, but once meiosis is complete, round and elongated spermatids seem to reactivate the expression of *Drd2/5, Adra1b*, and *Adrb2* (**Figure 1B**). In addition, we reported DRD1 expression in spermatogonia (67), whereas others found DRD2 in pre- and post-meiotic germ cells with predominant staining in spermatogonia (57). The α1-ADR immunodetection was observed from spermatogonia to elongated spermatids (75, 76), and specific *Adra1b* was reported in early spermatocytes and linked to subfertility (77). The expression data visualization available for the Human Testis dataset (**Figure 1C**) followed similar profiles to the mouse data, showing *DRD2* expression in undifferentiated spermatogonia (SSC and SG-1) and late spermatid stages (L-ST), and scattered high *DRD4* expression throughout all spermatogenic stages. Interestingly, the human data also showed specific profiles of α-ADRs transcripts, where α1-type *ADRA1A* and α2-type *ADRA2A* appear highly expressed in late spermatids and spermatocytes, respectively (**Figure 1C**).

The spermatogonia stages seem to be vulnerable for stress-induced epigenetic reprogramming since they reside outside the blood-testis barrier, being potentially exposed to all sources of catecholamines available in the testis, either plasmatic, or locally produced. The epigenetic status of spermatogonia shifts dramatically to either enter mitosis to self-renew or differentiate to type B and commit to meiosis. Undifferentiated spermatogonia have no expression of the repressive mark H3K9me2 nor the DNA methyltransferases *Dnmt3a2/3b*, whereas differentiated spermatogonia have increased H3K9me2 and upregulated *Dnmt3a2/3b* (78) which are maintained until round spermatid stage to make new DNA methylation patterns (7, 79). In line with this, we found that cocaine treatment altered *Dnmt3a/b* and *Tet1*, and increased 5-meC levels in mouse germ cells (65). Also, recent studies have shown that both DA *via* DRD1 and NE *via* β−ADRs induce alterations in DNA methylation patterns in the CNS (33, 80). Moreover, we have recently reported a U-shaped DRD1-dependent key testicular mechanism mediating cocaine-triggered increase in silent chromatin mark H3K27me3, decrease in active promoter mark H3K4me3, and increase in H4K16ac mark involved in histone to protamine replacement (66). Interestingly, activating H3K4me3 and reppresive H3K27me3 are bivalent marks simultaneously present at key promoters of embryonic developmental genes in both the spermatogonia and the retained nucleosomes in the mature sperm, carrying instructions for future embryo development (16, 17, 69).

The mouse mRNA profile shows that catecholamine inactivating enzymes *Comt/d1* and *Maoa/b* are detected throughout the entire spermatogenic process, suggesting that all germ cell types actively detoxify catecholamines. Also, spermatogonia and spermatids show *Th* and *Ddc* expression, indicative of dopaminergic identity. Moreover, spermatids seem to reactivate *Th* together with *Dbh* expression. This synthesizing enzymes profile suggest that male germ cells could be producing NE in the adluminal compartment, whereas DA could be the main catecholamine synthesized at the basal compartment by the undifferentiated SSCs. Recent groundbreaking work by Lepack et al. (81) showed that DA can associate directly with H3K4me3 to initiate an epigenetic marking of the chromatin called dopaminylation. They also demonstrated that histone dopaminylation in dopaminergic brain areas is involved in gene transcriptional programs that respond to cocaine consumption and craving (81). This work shows that DA synthesis could exert an epigenetic autocrine effect on its own, regardless of receptor activation, which sets a new paradigm from which to consider the possible consequences in germ cells H3K4me3 encoding.

In summary, we have put the spotlight on catecholamines as possible mediators between the stress response and epigenetic marks alterations during spermatogenesis. The expression of catecholaminergic components during germ cell maturation may point to these stress hormones as novel epigenetic regulators during spermatogonial and spermiogenic phases. Also, the expression of catecholaminergic components during spermiogenesis, when massive epigenetic events drive chromatin remodeling and nuclear compaction to produce mature spermatozoa, points to post-meiotic germ cells as a vulnerable window for stress-induced epigenetic reprogramming that should be further explored. Understanding the epigenetic regulation in paternal germ cells will pave the way to unravel the coding mechanisms in the transmission of the biological impacts of stress between generations.

## Author Contributions

CG and BG prepared the manuscript, conceptualized the idea, and revised the manuscript. All authors contributed to the article and approved the submitted version.

## Conflict of Interest

The authors declare that the research was conducted in the absence of any commercial or financial relationships that could be construed as a potential conflict of interest.

## Abbreviations

H, histone; PTMs, post-translational modifications; DA, dopamine; NE, nor-epinephine; TH, tyrosine hydroxylase; AADC or DDC, aromatic-L-aminoacid-decarboxylase; DBH, dopamine-beta-hydroxylase; PNMT, phenylethanolamine-N-methyltransferase (PNMT); DRD, DA receptor; ADR, adrenergic receptor; COMT, catabolic catechol-O-methyltransferase; MAO, monoamine oxidase; HPA, hypothalamic–pituitary–adrenal; CNS, central nervous system; BDNF, brain-derived neurotrophic factor.
